# A histidine kinase and a response regulator provide phage resistance to *Marinomonas mediterranea* via CRISPR-Cas regulation

**DOI:** 10.1038/s41598-021-99740-9

**Published:** 2021-10-18

**Authors:** Patricia Lucas-Elío, Luisa Raquel Molina-Quintero, Hengyi Xu, Antonio Sánchez-Amat

**Affiliations:** 1 Department of Genetics and Microbiology, University of Murcia, 30100 Murcia, Spain; 2 Department of Molecular Biosciences, Institute for Cellular and Molecular Biology, University of Texas at Austin, Austin, TX 78712 USA

**Keywords:** Microbiology, Molecular biology

## Abstract

CRISPR-Cas systems are used by many prokaryotes to defend against invading genetic elements. In many cases, more than one CRISPR-Cas system co-exist in the same cell. *Marinomonas mediterranea* MMB-1 possesses two CRISPR-Cas systems, of type I–F and III-B respectively, which collaborate in phage resistance raising questions on how their expression is regulated. This study shows that the expression of both systems is controlled by the histidine kinase PpoS and a response regulator, PpoR, identified and cloned in this study. These proteins show similarity to the global regulators BarA/UvrY. In addition, homologues to the sRNAs CsrB and CsrC and the gene coding for the post-transcriptional repressor CsrA have been also identified indicating the conservation of the elements of the BarA/UvrY regulatory cascade in *M. mediterranea*. RNA-Seq analyses have revealed that all these genetics elements are regulated by PpoS/R supporting their participation in the regulatory cascade. The regulation by PpoS and PpoR of the CRISPR-Cas systems plays a role in phage defense since mutants in these proteins show an increase in phage sensitivity.

## Introduction

Bacteria in their natural environments are exposed to many invading genetic elements such as phages and plasmids. These elements can constitute mechanisms of horizontal gene transfer conferring valuable attributes to the recipient cells, while they can also provoke cell death^1^. Amongst various bacterial defense mechanisms against invading elements, CRISPR-Cas systems are unique since they confer adaptative immunity to the cells. The CRISPR-Cas response is divided in three phases^2^. First, in the adaptation phase molecular memories of infection are formed by the acquisition of short segments of foreign nucleic acids, which are stored as ‘spacers’ in the CRISPR arrays^3^. Second, these CRISPR arrays are transcribed into precursor transcripts (pre-crRNA) which are subsequently processed into CRISPR RNAs (crRNA)^4^. The CRISPR-associated (Cas) proteins are the most important players in the third phase of interference. The Cas proteins, in general with nuclease activities, load the properly processed crRNAs to form a ribonucleoprotein (RNP). The functional CRISPR-Cas RNPs degrade the invading elements by using the crRNA to detect them by complementation with a short segment in the target nucleic acid sequence of the invading genetic elements^5^. There is an increasingly recognized diversity of CRISPR-Cas systems. So far, they are divided in two classes, 6 types (I to VI) and numerous subtypes, designed by a letter, depending on the *cas* genes and interference mechanism^6^.

One important element to be considered is the regulation of the expression of the CRISPR-Cas systems which is still poorly understood^7^. The acquisition of a CRISPR-Cas system, and in general of any other phage defense mechanism, can cause a metabolic burden to the cell, which needs to be balanced with the advantages offered for cell survival. In some cases, it has been observed that the expression of CRISPR-Cas systems is repressed and only induced after mutation in the repressing histone-like nucleoid-structuring (H-NS) protein^8,9^. Other CRISPR-Cas regulatory mechanisms described include quorum sensing, found in *Pseudomonas aeruginosa*^10^ and *Serratia*^11^, and an extracytoplasmic sigma factor (ECF) in *Myxococcus xanthus*^12^. Two-component regulatory systems (TCS) are composed of a membrane associated histidine kinase which senses an stimulus and transfers a phosphate group to a response regulator that controls the adaptative response to the stimulus^13^. TCS have been shown to regulate the expression of some CRISPR-Cas systems. The system composed by KinB and AlgB represses an I–F system in *Pseudomonas aeruginosa* during surface associated growth^14^. Recently, it has been shown that the Rcs (regulator of capsule polysaccharide synthesis) represses several CRISPR-Cas systems in *Serratia* when activated by stress factors^15^.

The marine bacterium *Marinomonas mediterranea* MMB-1 possesses two CRISPR-Cas systems of type I–F and III-B respectively^16^. The III-B system includes a fusion protein with three domains: Cas6, reverse transcriptase (RT) and Cas1. The RT domain is involved in the acquisition of spacers from RNA^17^, and the Cas6 domain is involved in crRNA processing^18^. Recent studies showed that the systems I–F and III-B cooperate in the defense against podoviruses, as two spacers in the I–F array targeting some of those phages could be used by both, the I–F and the III-B system^16^. This cooperation contributes to the survival of the bacteria when phages escape the I–F system, for example, by mutation in the Protospacer Adjacent Motif (PAM), since, unlike the type I–F system, the type III-B system does not require the PAM sequence to recognize the invader^16^.

The presence of two different CRISPR-Cas systems in *M. mediterranea* MMB-1, raises the question of if there is any common regulatory mechanism in their expression which could contribute to their cooperative activity. *M. mediterranea* MMB-1 has been also used as a model microorganism to study the expression of different oxidases such as the polyphenol oxidases (PPOs) tyrosinase and laccase^19,20^, as well as L-amino acid oxidases with a quinone cofactor^21^. PpoS, for Polyphenol oxidase Sensor kinase, is a hybrid histidine kinase first described in the mutant strain T103 (PpoS^−^) which shows lowered expression of the laccase and the tyrosinase involved in melanin synthesis^22^. Later, it was shown that the expression of LodA, a lysine epsilon-oxidase with a quinone cofactor, is regulated at the transcriptional level by PpoS^23^. PpoS shows similarity to *Escherichia coli* BarA which forms part of a two-component regulatory system with its cognate response regulator UvrY^24^. Orthologs of the BarA/UvrY system have been characterized in other Gram negative bacteria, such as the GacS/GacA in *Pseudomonas*^25^. These systems have been described to form central hubs within complex regulatory networks and have mainly been studied in pathogens for their regulation of virulence factors such as exoenzymes or toxins, along with stress response, motility, quorum sensing, biofilm formation and many functions of the carbon metabolism^26^.

According to the NCBI Conserved Domain Search^27^ PpoS shows the conserved domain PRK11107, hybrid sensory histidine kinase BarA, which is characteristic of hybrid histidine kinases (HK) that form part of phosphorelay systems. Hybrid HKs autophosphorylate and then shuttle the phosphoryl group through different domains until it is transferred to a separate response regulator protein. Response regulators control many different cellular processes, and exert their regulatory action by controlling the expression of other genes at the transcriptional level. Response regulators like GacA recognize the denominated GacA box (TGTAAGN_6_CTTACA) located upstream of the genes to be regulated^28^. Generally, BarA/UvrY like systems activate the transcription of CsrA binding sRNAs. CsrA is a regulatory protein that post-transcriptionally represses target genes. The formation of the CsrA-sRNA complex sequesters CsrA from binding to its target genes^28^. While CsrA gene is relatively conserved, the sRNAs genes are not conserved in their gene sequence and gene copy numbers, which makes difficult the identification of the CsrA-sRNA like components. For example, *E. coli* has only one sRNA gene, but in *Pseudomonas fluorescens* and *P. aeruginosa* two of them, named RsmY and RsmZ, have been described^25,26^.

The aim of this study has been to gain insights into the process of regulation of CRISPR-Cas systems in *M. mediterranea*. First, in addition to the PpoS histidine kinase, we identified several novel regulatory elements with homology to those in BarA/UvrY systems. By using transposon mutagenesis, we identified a response regulator (PpoR) with similarity to UvrY. The similarity in the transcriptomic profile and phenotype of the strains mutated in PpoS and PpoR suggests that both proteins may participate in the same regulatory cascade. Other elements of the regulatory cascade such as CsrA1 and two sRNAs (CsrB and CsrC) homologues have been also detected by bioinformatics analysis in this study. RNA-Seq analyses have revealed that PpoS and PpoR regulate, at the transcriptional level, the expression of the two *cas* operons in *M. mediterranea*. This regulatory mechanism takes place in the absence of phages and generates a physiological state that facilitates the resistance to the phages as revealed by the increased sensitivity to them of PpoS^−^ and PpoR^−^ mutants.

## Results

### Identification of a response regulator, PpoR, affecting a diversity of physiological processes in *M. mediterranea*

**Figure 1:**
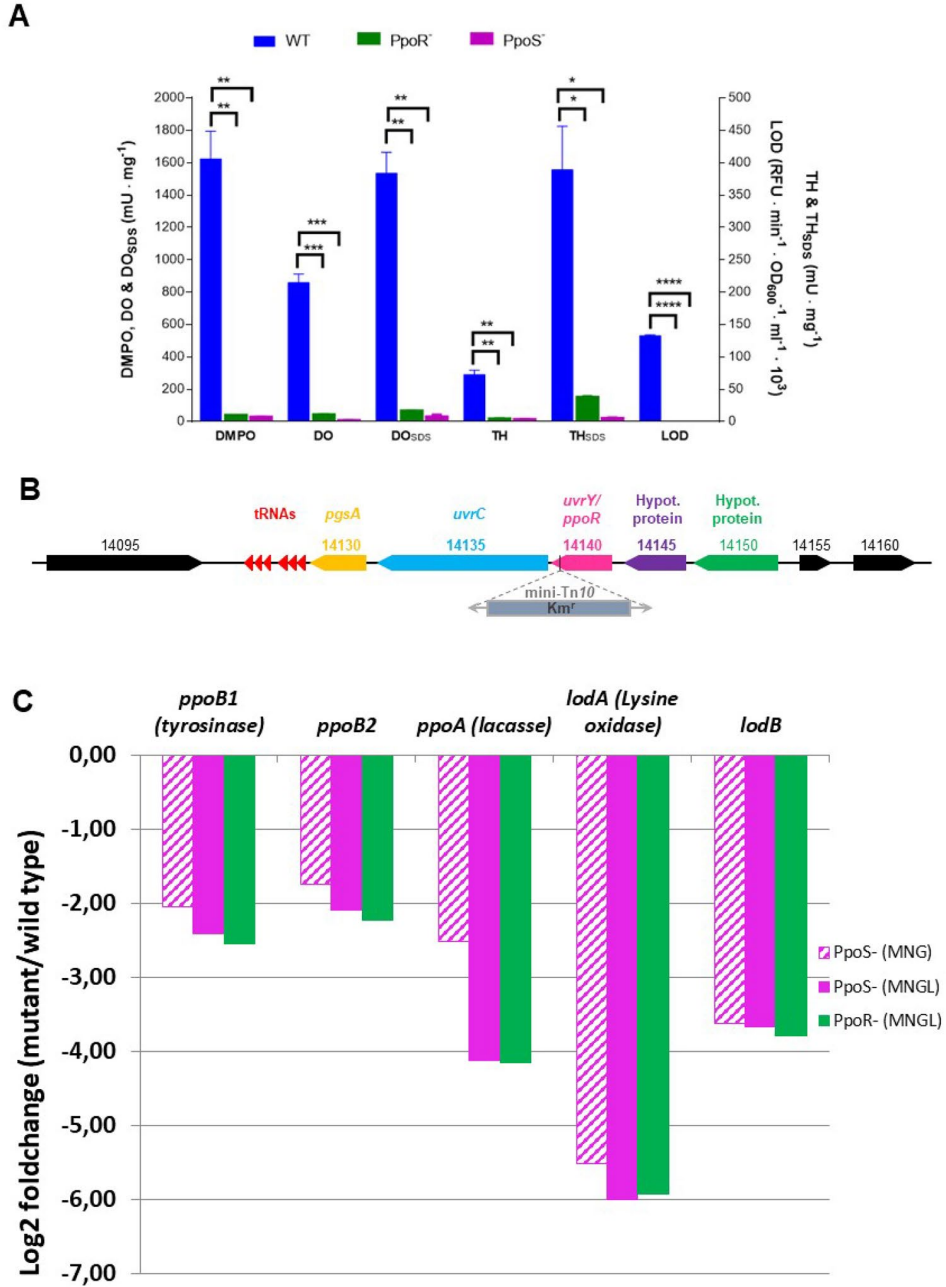
Identification of the response regulator PpoR. (**A**) Oxidase activities in *Marinomonas mediterranea* strains: DMPO (Dimethoxyphenol oxidase, characteristic of laccase), DO (L-dopa oxidase), TH (Tyrosine hydroxylase) and LOD (ε-lysine oxidase). The subscript SDS indicates activity in the presence of SDS which is characteristic of the tyrosinase. Blue bars, wild type strain (MMB-1R); green bars stand for strain PpoR^−^; and magenta bars for strain PpoS^−^. Error bars represent means ± s.d, (n = 2). *P < 0.05; **P < 0.01. ***P < 0.001, ****P < 0.0001 according to one-way ANOVA followed by Tukey's post test. (**B**) Genomic region surrounding the site of insertion of the transposon in strain T102. Marme_RS14095, MARME_RS14145 and MARME_RS14150 encode hypothetical proteins. MARME_RS14130 encodes a CDP-diacylglycerol glycerol-3-phosphate 3-phosphatidyltransferase (PgsA). MARME_RS14135 shows similarity to *uvrC*. (**C**) PpoR and PpoS mutations show a very similar effect on the transcriptomic regulation in MNGL and MNG media, determined by RNA-Seq, of the oxidase genes in *M. mediterranea*: *ppoB1* (tyrosinase), *ppoB2* (copper chaperone), *ppoA* (laccase), *lodA* (ε-lysine oxidase), *lodB* (flavoprotein post-transcriptionally processing LodA).

The hybrid histidine kinase (HK) PpoS regulates in *M. mediterranea* different enzymatic activities such as laccase, tyrosinase (involved in melanin synthesis) and ε-lysine oxidase^22,23^. Since PpoS bears similarity to *Escherichia coli* BarA which is a global regulator affecting many different processes, we hypothesized that it could also control CRISPR-Cas expression in *M. mediterranea*. Our first goal was to identify the cognate response regulator of PpoS in *M. mediterranea*. Bioinformatic analysis of *M. mediterranea* MMB-1 genome with the P2CS (Prokaryotic 2-Component Systems) server^29^ revealed 57 putative response regulators and 40 histidine kinases, making it difficult the prediction of the cognate regulator associated to PpoS. In order to detect it experimentally, we hypothesized that a mutant with a similar phenotype to strain T103 (PpoS^−^) could be of interest in this study. The screening of several thousand colonies generated by transposon mutagenesis of the wild type strain, revealed an strain named T102, which was amelanogenic, similarly to what had been observed in strain PpoS^−^^30^. Furthermore, the similarity to strain PpoS^−^ was also revealed by the analysis of several enzymatic activities. Regarding PPO activities, the activities characteristic of the tyrosinase (TH_SDS_ and DO_SDS_) and of the laccase (DMPO) of the two mutant strains showed activity levels reduced for more than 90% in comparison with the wild type strain (Fig. 1A). No lysine oxidase (LOD) activity was detected neither in strain T102 nor in strain PpoS^−^, even when inoculated in medium MNGL. This medium differs to MNG in the addition of 3 mM L-lysine which is an inductor of LOD activity and, accordingly, in this medium this activity is high^23^ (Fig. 1A). These results suggest that the product of the gene mutated in strain T102 may function in the same regulatory pathway as PpoS.

The gene mutated in strain T102 has been identified in this study by genome walking as detailed in material and methods. The gene mutated by transposon insertion corresponded to the locus MARME_RS14140 annotated in the *M. mediterranea* genome as a “response regulator”. The insertion interrupts the gene at position 357 of 642 leaving a truncated gene. BLAST analysis using the product of MARME_RS14140 revealed that it encodes a response regulator similar to UvrY (45.9 identity and 68.8% similarity) of the *E. coli* BarA/UvrY system and to GacA (50.9 identity and 71.1% similarity) of the *Pseudomonas* GacS/GacA two-component system^28^. According to the bioinformatic analysis of the product of this gene in Pfam databases^31^, this protein contains from amino acids 4 to 116, a response regulator receiver domain which would receive the signal from a sensor partner protein. There is also a DNA binding effector domain typical of bacterial regulatory proteins, LuxR family, from amino acids 148 to 204. This kind of proteins controls the transcription of the target genes by binding to DNA through their DNA binding domain^32^. The product of MARME_RS14140 has been named as PpoR, standing for polyphenol oxidases response regulator. Accordingly, the mutant T102 will be named from now on as PpoR^−^.

The bioinformatic analysis of the genomic sequence surrounding *ppoR* revealed the existence of several loci in the same reverse orientation as *ppoR* (Fig. 1B). The two genes upstream of *ppoR*, MARME_RS14145 and MARME_RS14150, encode hypothetical proteins. Regarding the genes downstream *ppoR*, MARME_RS14135 shows similarity to *uvrC*, a gene encoding a hypothetical endonuclease part of the enzymatic complex UvrABC that repairs DNA. MARME_RS14130 encodes a CDP-diacylglycerol glycerol-3-phosphate 3-phosphatidyltransferase (PgsA), a protein involved in lipid metabolism. The contiguous disposal of genes homologous to *uvrY*, *uvrC* and *pgsA* respectively is conserved in other bacteria including *E. coli*. In this microorganism, it has been observed that *pgsA* does not form part of the same transcriptional unit as the other two genes^33^. RT-PCR analysis has been performed to test whether in *M. mediterranea ppoR* and *uvrC* also belong to the same transcriptional unit, being observed that this is the case with no evidences of any other gene forming part of it (Supplementary Fig. S1). Since PpoR^−^ is a mutant generated by transposon insertion it could not be ruled out some downstream effect on the expression of *uvrC*, the second gene in the operon, which, by similarity with other systems, could affect the observed phenotype^34^. To study this possibility, a series of vectors were constructed: pRU contains the whole operon (*ppoR-uvrC*); pR only the *ppoR* gene and pU contains the *uvrC* gene. pC is the control plasmid without any insert. These plasmids were mobilized into PpoR^-^ strain. It was first observed that melanin synthesis was restored when the plasmid pR (*ppoR)* was mobilized. The same effect was observed by mobilizing the plasmid pRU with the whole operon. On the contrary, melanin synthesis was not restored by cloning only *uvrC*. Enzymatic assays revealed that all PPOs activities as well as the lysine oxidase activity were recovered to wild type levels by mobilizing pR (*ppoR)* or pRU (*ppoR-uvrC)*, while the mobilization of pU (*uvrC)* alone had no effect (Supplementary Fig. S2). These data indicated that the phenotype observed in strain PpoR^-^ was the result of the mutation in *ppoR.*

To analyze the regulatory mechanism exerted by PpoR and PpoS the mRNA levels of all oxidase coding genes were determined by RNASeq. The results obtained showed that all of them were down regulated in the PpoS^−^ and PpoR^-^ mutant strains in media MNG and MNGL (Fig. 1C). Previous studies of our group revealed that the genes of the operon *lod*, coding for an ε-lysine oxidase are regulated at the transcriptional level by PpoS. This regulation was demonstrated by two different methods, qRT-PCR and transcriptional fusions of the *lod* promoter to the *lacZ* gene^23^. RNA-Seq also offered the same results for the two genes of the operon with a Log2 fold change of − 3.71 for *lodB* and − 6.04 for *lodA* referred to the wild type strain, which constitutes a validation of the results obtained by the RNA-Seq method. The comparison of the transcriptomic levels of all coding genes in the strains mutated in *ppoS* and *ppoR* revealed that in both strains 98.46% of the genes showed a similar level of expression, with the differences in their level of expression below 2 fold. Only 1.17% of the genes showed higher expression in strain PpoR^−^ than in strain PpoS^−^, and 0.43% showed a lower level of expression. In comparison with the wild type strain, in strain PpoS^−^ the 5.95% of the genes were overexpressed (> twofold change) and the 8.83% were repressed (< twofold change). In the PpoR^−^ strain the values were 8.44% overexpressed and 9.14% repressed. Notably, most of the genes overexpressed or repressed in the conditions analyzed were identical in the two genetic backgrounds (Supplementary Fig. S3). Although in this study it has not been demonstrated that PpoS phosphorylates PpoR, the transcriptomic analysis strongly suggests that both proteins participate in the same regulatory cascade. Nevertheless, it cannot be ruled out the interaction of PpoR or PpoS with other histidine kinases or response regulators due to the cross-talk between elements of TCS^35^.

### PpoS and PpoR control the expression of a CsrA homologue and two sRNAs homologous to CsrB and CsrC

PpoS and PpoR bear similarity to two-component regulatory systems (TCS) such as BarA/UvrY. These systems show some conserved elements. CsrA is a protein involved the post-transcriptional repression of many cellular processes. This protein and its orthologs are global regulators, acting primarily as repressors by binding mRNA targets, affecting their translation, stability and abundance^36^. TCSs similar to BarA/UvrY transcriptionally regulate the expression of some sRNAs. These sRNAs are able to bind and sequester CsrA and its orthologs thereby alleviating  the repression of target mRNAs^28^. Bioinformatic analyses using published protocols^37^ predicted the presence of two sRNAs which could be regulated by PpoR/PpoS. One of them, CsrB, is encoded in the intergenic region between MARME_RS03610 and MARME_RS03615. The second one, CsrC, is encoded upstream of MARME_RS17890. Both sRNAs show the typical elements of CsrA regulating sRNAs (Supplementary Figs. S4 and S5). Their sequences contain a GacA box (TGTAAGN_6_CTTACA) recognized by UvrY and its orthologous proteins. The analysis of their coding sequences revealed the presence of many CsrA binding motifs, with the sequence ANGGA/AGGA, which were confirmed by the prediction of their secondary structure by the mfold web server (http://www.unafold.org/). Finally, at the end of the sequence there is a Rho-independent transcription terminator (Supplementary Figs. S4 and S5). All these features strongly indicate that the detected sequences correspond to CsrA regulating sRNAs.

In order to identify proteins similar to CsrA, which are conserved in BarA/UvrY regulatory cascades, we performed a BLASTp search using as a query the sequence of *E. coli* CsrA. Two orthologues to CsrA were found in the *M. mediterranea* genome. One is the product of the gene MARME_RS03780 with 81% identity and 93% similarity to *E. coli* CsrA and it has been named as CsrA1. The second protein with similarity to CsrA, named CsrA2, encoded by MARME_RS09140, showed lower identity (64%) and similarity (87%). In addition, the levels of expression of this second homologue were very low in all the conditions assayed. For instance, in MNG medium the normalized counts of *csrA1* were 1208.17 while for *csrA2* were 36.06. Accordingly, CsrA2 was not further analyzed in this study. The similarity of *M. mediterranea* CsrA1 to *E. coli* CsrA is stressed by the genomic location of their genes. Both of them are located downstream of an alanyl-tRNA synthetase, an aspartate kinase and upstream of some tRNAs^38^. Interestingly, upstream of the gene coding for *M. mediterranea* CsrA1, a sequence (TGTAGGGGAATTCTTACA) with high similarity to the GacA box was detected. In this sequence all the residues, but one, of the GacA box are conserved (underlined). This observation suggested a direct regulation of the *csrA1* gene in *Marinomonas mediterranea* by the TCS.

**Figure 2:**
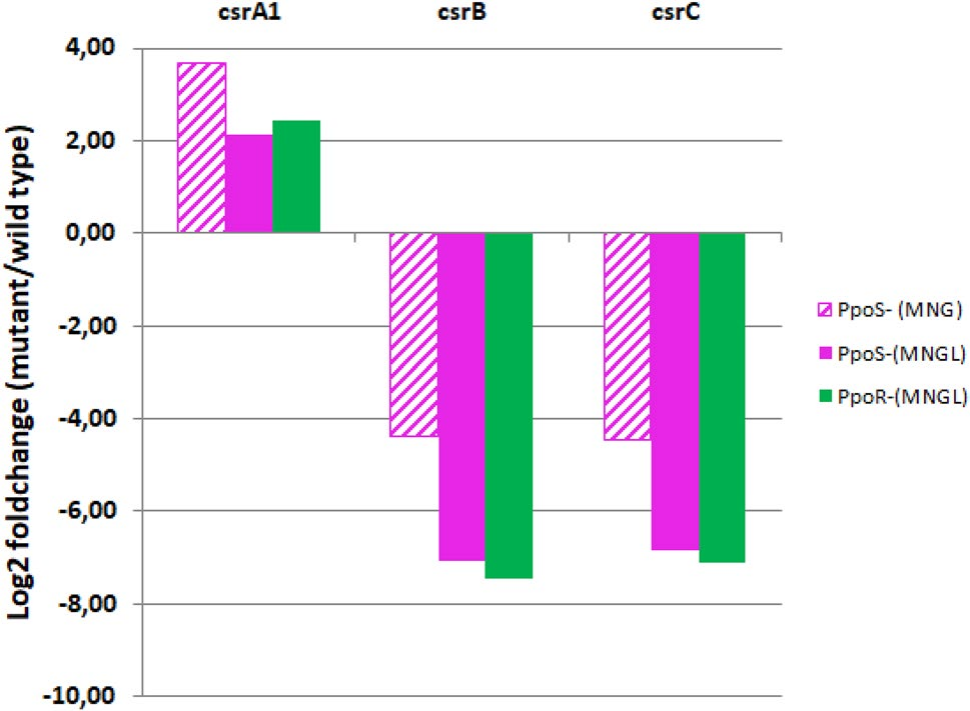
Transcriptomic regulation of *csrA1*, and the sRNAs *csrB* and *csrC* by PpoS and PpoR. The relative expression of these genes in the PpoS^−^ (magenta bars) and PpoR^−^ (green bars) mutants was determined in comparison with the wild type strain in medium MNGL*.* Data for strain PpoS^−^ in MNG medium are also included.

To study the regulation of the expression of those sRNAs and evaluate their possible participation in the PpoS/R cascade, their transcriptomic levels in PpoS^−^ and PpoR^-^ mutant strains were analyzed in comparison with the WT strain. The coverage plots of the reads in those analyses were in agreement with the bioinformatics predictions of the sRNAs. In fact, in comparison with the surrounding genes, both sRNAs showed a high level of expression (Supplementary Fig. S6). In agreement with the presence of the GacA box, the regulation of the expression of the sRNA by PpoS and PpoR could be detected in all the conditions analyzed since their levels in mutants in those proteins decreased greatly in comparison with the WT strain (Fig. 2). These results indicate the positive regulation by PpoS and PpoR of the expression of the detected sRNAs. Contrary to the decrease observed for *csrB* and *csrC*, the transcriptomic analyses revealed that in the PpoS^−^ and PpoR^-^ mutant strains the expression of *csrA1* increased (Fig. 2). These results strongly suggest a direct repression at the transcriptomic level of *csrA1* by the regulatory cascade and further supports the hypothesis of PpoS and PpoR belonging to the same regulatory cascade.

**Figure 3:**
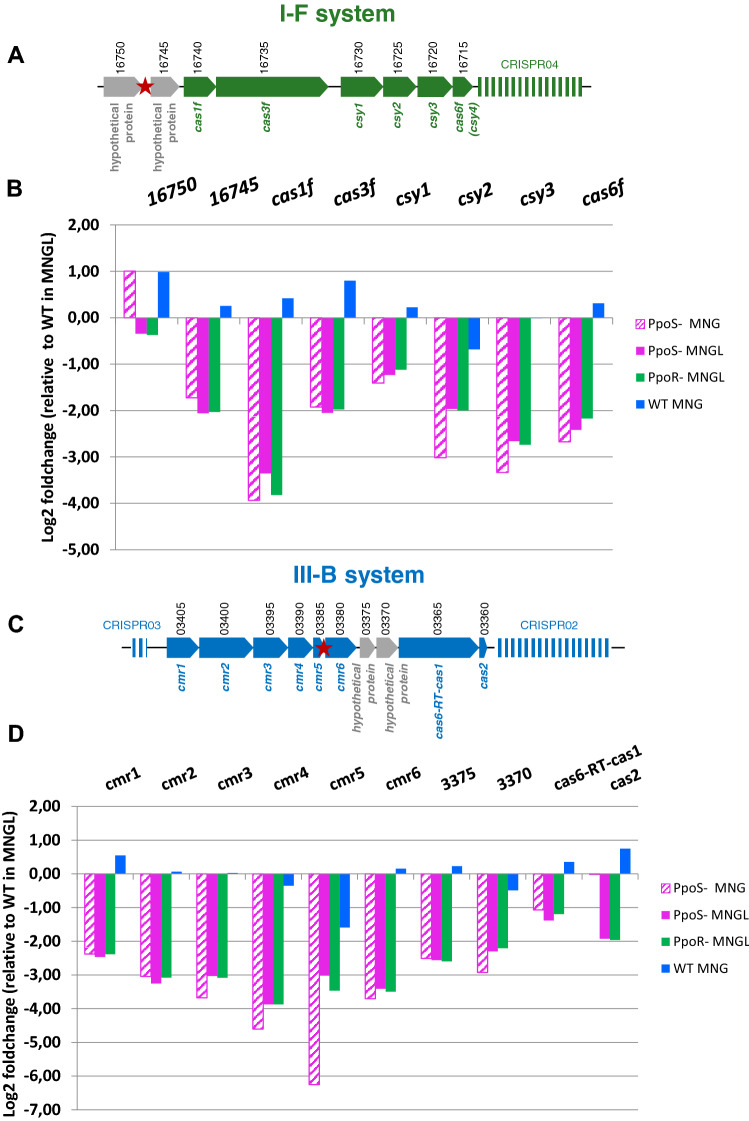
Regulation of CRISPR-Cas expression in *M. mediterranea* by the histidine kinase PpoS and the response regulator PpoR. (**A**) Schematic representation of the I–F CRISPR-Cas system. The CsrA1 potential binding site is marked with a red star. (**B**) Transcriptomic levels of the I–F CRISPR-Cas system in different genetic backgrounds and MNGL or MNG media relative to the WT levels in MNGL. (**C**) Schematic representation of the III-B CRISPR-Cas system. The CsrA1 potential binding site is marked with a red start. (**D**) Transcriptomic levels of the III-B CRISPR-Cas system in different genetic backgrounds and MNGL or MNG media relative to the WT levels in MNGL. The genes in grey are not present in all similar CRISPR-Cas systems.

The in-silico prediction of possible CsrA1 binding sites using the program CSRNA_TARGET^39^, revealed two potential targets of interest since they are in the CRISPR-Cas regions of both the I–F and III-B systems in *M. mediterranea* (Fig. 3, Supplementary Fig. S7).

### The histidine kinase PpoS and the response regulator PpoR regulate the expression of the two CRISPR-Cas systems in *M. mediterranea*

The expression of the CRISPR-Cas systems in *Marinomonas mediterranea* (Fig. 3A,C) was analyzed by RNA-Seq in the PpoS^−^ and PpoR^−^ strains in comparison with the wild type strain (Fig. 3). Two genes coding for hypothetical proteins, a methyltransferase and a WYL-domain containing protein, respectively, located upstream of the canonical genes of the I–F systems were included in the analysis because the CsrA binding site detected in this study is located between these two genes (Fig. 3A, Supplementary Fig. S7). As previously discussed, the medium MNGL induces the expression of some oxidase enzymes. On the contrary, the expression of the CRISPR-Cas genes showed very similar values in the two cultures of the wild type strain in media MNG and MNGL (Fig. 3B,D). This result agrees with the observation that  *M. mediterranea* shows the same sensitivity to phage CB5A in both media (data not shown). It was observed that in comparison with the wild type strain, both, the I–F and the III-B CRISPR-Cas systems, were downregulated in the mutant strains PpoS^−^ and PpoR^−^ analyzed in this study (Fig. 3B,D). In the case of the I–F system, the gene MARME_RS16745 is regulated similarly to the *cas* genes, suggesting that it may belong to the same transcriptional unit. It is important to indicate that the down regulation of the CRISPR-Cas genes was always in the same range regardless of the mutant strain or media analyzed in this study. Statistical analyses have shown that the normalized counts of the WT strain in the two media show significant differences with the counts in the mutant strains (Supplementary Fig. S8). This robustness in the results suggests that the regulation of the CRISPR-Cas systems by PpoS and PpoR is conserved across a range of growth conditions.

### Mutations in PpoS and PpoR increase phage sensitivity

**Figure 4:**
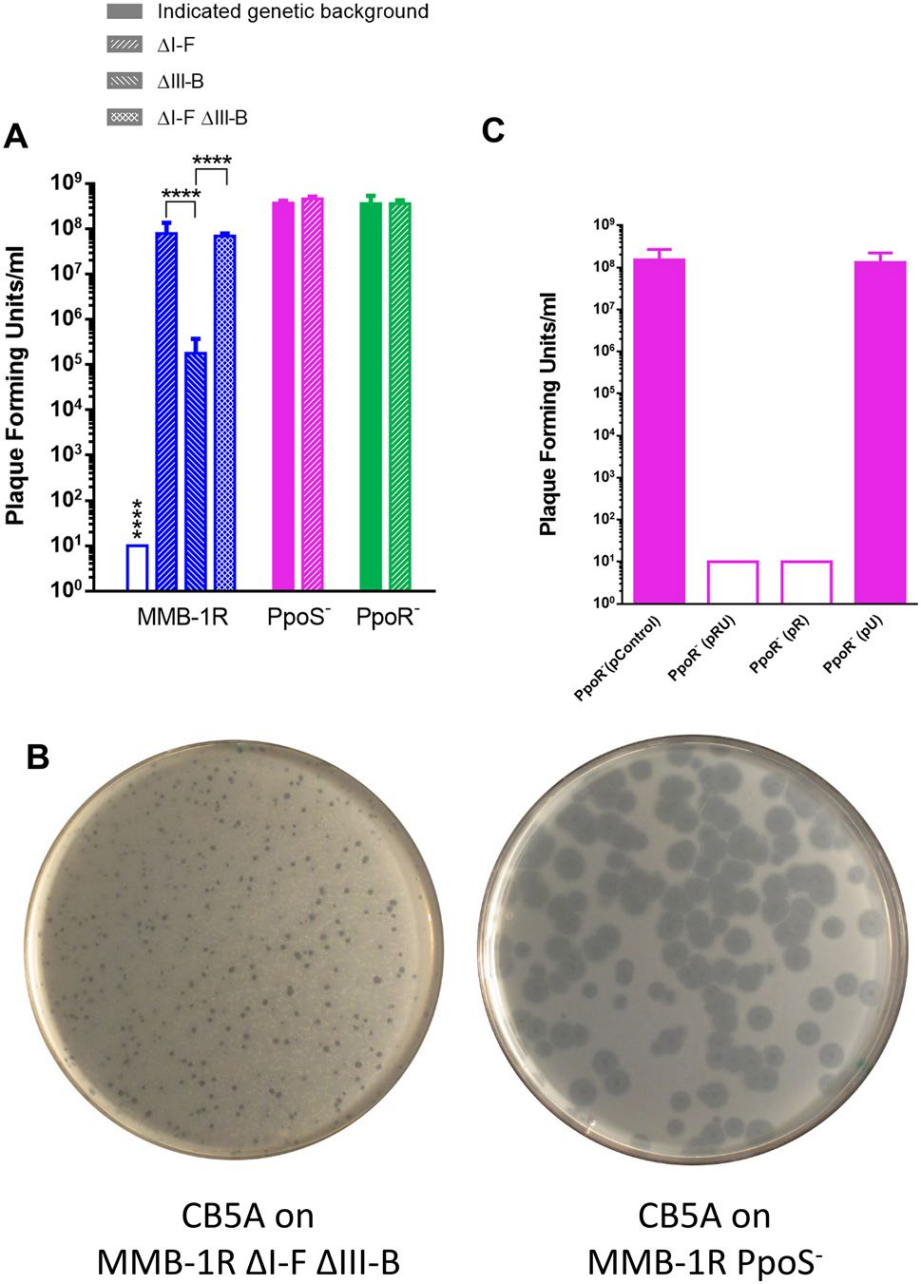
Susceptibility of different *M. mediterranea* strains to phage CB5A. (**A** and **C**) Plaque forming units in double layer assays in MNG medium. Empty columns indicate the detection limit when no phage plaques were detected in the assays at the higher phage concentration. (**A**) Only the data of wild type strain (MMB-1R) and the ΔIII-B strains showed statistical difference among themselves and with all the other samples. Error bars represent means ± s.d (n = 3) ****P < 0.0001 according to one-way ANOVA followed by Tukey's post-test. (**C**) No statistical difference was observed between PpoR^-^ control and PpoR^-^ (pU, containing the *uvrC* gene). No plaques were observed in lawns of PpoR^-^ (pR, containing *ppoR*) or PpoR^-^ (pRU, containing *ppoR-uvrC*) (**B**) CB5A plaques on lawns of the ΔI–F ΔIII-B strain (left) and PpoS^−^ (right).

To evaluate the effect of the regulation of the CRISPR-Cas systems by PpoS and PpoR in the sensitivity to the podovirus CB5A, the sensitivity of strains PpoS^−^ and PpoR^−^ was studied in comparison with the wild type strain and the mutants in CRISPR-Cas systems generated in this genetic background which were previously described by our group^16^. In agreement with previous results, the deletion of the I–F system increased the sensitivity to phage CB5A in comparison with the wild type, since this deletion included the array containing the two spacers used by both, the I–F and the III-B systems to defend against the infection by the phage^16^ (Fig. 4A). In the medium MNG used in these experiments, no phage plaques were detected against the wild type strain, while an increase in the sensitivity of the ΔI–F strain of several orders of magnitude was observed, which is higher than the increase previously described in complex medium^16^. Under the conditions of the assay, the deletion of the III-B system alone increased the sensitivity to the phage more than 1000-fold (Fig. 4A). This observation is in agreement with previous results of our group showing that although the III-B system does not contain in its CRISPR array spacers against the podoviruses, it can use the spacers in the I–F system^16^.

The PpoS^−^ and PpoR^−^ mutants showed a sensitivity comparable with the one observed in the strains with a deletion of the I–F or the I–F plus III-B operons (Fig. 4A). The small differences in plaque counts observed were not statistically significant. There was a difference in plaque sizes, the ΔI–F and ΔIF ΔIIIB mutants showed a plaque size smaller than the PpoS and PpoR mutants, (Fig. 4B). The bigger size of the plaques could facilitate their detection explaining the slightly higher counts in the regulatory mutants. To further study this sensitivity, the deletion of the I–F system was introduced in the genetic background of the mutants PpoR^−^ and PpoS^−^ , generating the double mutants PpoS^−^ ∆I–F and PpoR^−^ ∆I–F. The increase in sensitivity observed in comparison with the single mutants was not significant (Fig. 4A). Overall these results indicate that there is no difference in phage sensitivity in the wild type strain with deletion of the CRISPR-Cas systems, the PpoS^−^ and PpoR^−^ mutants and the double mutants in CRISPR and the regulatory proteins. These results indicate that the regulation of the expression of the CRISPR-Cas systems by PpoS and PpoR is the main factor involved in the increased sensitivity to phage CB5A of the mutant strains PpoS^−^ or PpoR^−^ in comparison with the wild type strain.

The sensitivity of the PpoR^−^ mutant strains complemented with pC, an empty vector, or with vectors pRU, containing the whole operon (*ppoR-uvrC*), pR in which *ppoR* was cloned or pU with only *uvrC* was assayed. It was observed that the mobilization to the PpoR^−^ mutant of *ppoR*, alone or in combination with *uvrC*, restored the resistance to the phage up to the wild type levels (Fig. 4C). On the contrary the mobilization of *uvrC* had no effect (Fig. 4C). These results show that the possible downstream effect on *uvrC* expression, has no role in the sensitivity of the PpoR^−^ mutant to phage CB5A and that this increased sensitivity is the result of the *ppoR* mutation.

## Discussion

Most of the research on CRISPR-Cas systems has been related to their mechanism of action and their biotechnological applications. Comparatively, much less is known about the interplay of CRISPR-Cas with other cellular processes such as cell metabolism and other defense mechanisms, including additional CRISPR-Cas systems. The study of the regulation of CRISPR-Cas systems could offer insights into those processes. The two-component system BarA/UvrY is a global regulator in *E. coli* controlling the expression of many different processes such as motility, metabolism and virulence. Within this regulation takes place the activation of the transcription of sRNAs (CsrB and CsrC) which sequester the RNA binding protein CsrA. This protein regulates gene expression, acting primarily as a repressor, by binding mRNA targets, affecting their translation, stability and abundance^36^. This regulatory circuit is present in many *Gammaproteobacteria* where it receives different names in different species^28^. In this study it is was shown that orthologous to those genetic elements are also present in the marine bacterium *M. mediterranea.* Previous studies of our group had shown that in *M. mediterranea* the composite histidine kinase PpoS regulates the expression of a laccase, a tyrosinase (affecting melanin synthesis) and an ε-lysine oxidase with a quinone cofactor^22,23^. We have now detected PpoR, the orthologous of the *E. coli* response regulator UvrY (GacA in *Pseudomonas*). The high similarity between the PpoR^-^ mutant and the PpoS^−^ mutant at both, phenotypic and transcriptomic level, suggests that they must participate in the same route. CsrA and the sRNAs orthologues have also been identified, as well as putative regions in the CRISPR-Cas operons which could be targets of CsrA.

The main observation in this study is that the mutation in PpoS or PpoR determines the repression of the expression of the two CRISPR-Cas systems of *M. mediterranea,* indicating that they are under the control of the PpoS/R system. We have also identified other genetic elements which, by similarity with the functionality of systems in other bacteria, could participate in the regulatory cascade. The RNAseq analyses support this possibility since it was observed that PpoS and PpoR mutants show an over-expression of CsrA1 and a down-regulation of the sRNAs CsrB and CsrC. Altogether, these observations are compatible with a regulatory mechanism in which, once the stimulus is received by a cascade including PpoS and PpoR, the induced sRNAs would sequester CsrA1 and thus alleviate its repression on the CRISPR-Cas system, allowing their expression. An important novelty in our model is that we have observed the repression of *csrA1* transcription exerted by PpoS and PpoR (Fig. 2). That repression can be mediated by the presence of a GacA binding motif upstream of the sequence of *csrA1.* This additional regulatory mechanism would cooperate with the action of the sRNAs sequestering any expressed CsrA1, to alleviate the effect on the genes repressed by this protein. Experiments aimed at the construction of a strain with a deletion of CsrA1 to study its role in the regulatory cascade have not been successful so far (data not shown). This could be related to the fact that CsrA has proven to be essential in several microbial strains^28^.

The regulation of the *M. mediterranea* CRISPR-Cas systems by PpoS and PpoR is key in the defense against phages as revealed by the fact that mutants in these proteins show an increased sensitivity to phage infection. There is no statistical difference in the sensitivity to phage CB5A between the mutants in PpoS or PpoR to the one observed in the mutant with deletion of the two CRISPR-Cas systems (Fig. 4). Furthermore, the deletion of the I–F system, and hence of the spacers used by both CRISPR-Cas systems, in the PpoS/PpoR mutant strains did not increase the sensitivity to the phages. Overall, these results indicate that, in the experimental conditions used in this work, the regulation by PpoS and PpoR of the I–F and III-B systems is the main cause of the increased sensitivity of PpoS^−^ and PpoR^−^ mutants to phages.

As far as we know, this is the first description of a BarA/UvrY orthologous system regulating the expression of CRISPR/Cas or any other mechanism of defense against phages. Other TCSs have been also shown to control CRISPR-Cas systems. The KinB-AlgB system regulates alginate biosynthesis and represses a type I–F CRISPR-Cas system during surface associated growth in *Pseudomonas aeruginosa*^14^. This regulation determines changes in the sensitivity to the phages that are dependent on the spacer used by the CRISPR-Cas system, and was not higher than one order of magnitude in any case^14^. The *Serratia* Rcs system behaves in an opposite way to the *M. mediterranea* PpoS/R system since it represses the expression of two CRISPR-Cas systems of type I–F and III-A, respectively, which was inversely correlated with an increase of surface immunity against different phages under stress conditions^15^. This lowered CRISPR-Cas expression under stress conditions has been proposed to facilitate the process of horizontal gene transfer^15^. Interestingly, similarly to the *P. aeruginosa* system, that repression takes place in conditions in which there is synthesis of capsular material.

The regulation by PpoS and PpoR, as well as the other processes described above, takes place in the absence of a phage challenge. In this regard, it bears similarity to the regulation of CRISPR-Cas expression by quorum sensing (QS) in which higher levels of expression of CRISPR-Cas proteins are observed in conditions of high cell density, when the cells are more vulnerable to phage attack^7,11^. In *M. mediterranea* the regulation by PpoR and PpoS affects many different processes. Cells with mutations in those regulatory proteins are more sensitive to phages and are down-regulated in the synthesis of melanins and the expression of laccase and ε-lysine oxidase. The induction of *M. mediterranea* oxidase activities takes place at the stationary phase of growth^22,23^. This could take place mediated by CsrA1, since this protein regulates the transition from exponential to stationary phase of growth in many different microorganisms^40^. The expression of all the traits regulated by PpoS^−^ and PpoR^−^ could be important for the survival of the cells in conditions of high cell density such as those generated at the stationary phase of growth. In these conditions, the expression of the two CRISPR-Cas systems in *M. mediterranea* would offer the possibility of defending against a wide variety of infecting genetic elements. This defense will be enhanced since the systems I–F and III-B cooperate in the defense against phages as the I–F crRNAs can be used by both, the I–F and III-B systems. This redundancy mechanism avoids phage escaping the I–F system, for example by mutation in the PAM sequence^16^. In consequence, their co-regulation would greatly improve their survival options.

In this manuscript we identify a regulatory mechanism controlling the expression of two CRISPR-Cas systems in the marine bacterium *M. mediterranea.* This system is homologous to the *Escherichia coli* BarA/UvrY system which is widely distributed in gammaproteobacteria. This and other studies are revealing that the CRISPR immunity is regulated by different environmental conditions which would play an important role in terms of the balance of cost/benefits associated to CRISPR expression.

## Materials and methods

### Bacterial strains, plasmids and growth conditions

All bacterial strains, plasmids and primers used in this work are listed in Table 1. *Marinomonas mediterranea* strains were cultured at 25 °C in the marine media MM2216 (Difco), MMC^41^, MNG or MNGL, depending on the experiment^23^. When culturing in liquid media, flasks were incubated on an orbital shaker at 130 rpm. *E. coli* was cultured in Miller’s LB medium (Pronadisa), at 37 °C and 200 rpm, except for the culture used for conjugation that was grown at 60 rpm to avoid sex pili breakage^20^. When required, the media were supplemented with the appropriate antibiotics (Sigma).

**Table 1:** Strains, plasmids and primers used in this study. The phenotypes described for the strains inside the brackets mean: PPO^+/−^ (decrease of all PPO activities), MEL^+/−^ (decrease of melanin synthesis), LOD- (loss of lysine oxidase activity). Primers are marked with (d) meaning direct if they hybridize with the template strand, or (r) from reverse if they hybridize with the coding strand.

Strains	Genotype and/or relevant phenotype	References
***M****. ****mediterranea***		
MMB-1^ T^	Wild type strain, Rif^s^, Gm^s^	^19^
MMB-1R	MMB-1, spontaneously Rif^r^	^20^
MMB-1R ΔI–F	MMB-1R, Δ CRISPR I–F	^16^
MMB-1R ΔIII-B	MMB-1R, Δ CRISPR III-B	^16^
MMB-1R ΔI–F ΔIII-B	MMB-1R, Δ CRISPR I–F and Δ III-B	^16^
T103 ( PpoS^−^)	MMB-1R *ppoS*::Tn*10* Km^r^, [PPO^+/−^, MEL^+/−^, LOD^-^]	
T102 (PpoR^-^)	MMB-1R *ppoR*::Tn*10* Km^r^, [PPO^+/−^, MEL^+/−^, LOD^-^]	^30^
T103 ΔI–F	T103, Δ CRISPR I–F	This study
T102 ΔI–F	T102, Δ CRISPR I–F	This study
***E****. ****coli***		
S17-1(λ*pir*)	Km^r^::Tn*7* Tp^r^ Sm^r^ *recA ths hsdRM*^+^; λ*pir* phage lysogen RP4::Mu::Km Tn*7*	^49^
**Primers**	**Sequence**	
Adaptor 1	5′-GTCATACGACGGTACCTGCAGAATTCTCTAGAAGCTTCCCGGGCTGGT-3′	
Adaptor 2	5′-(Phos)ACCAGCCC(AmC3)-3′	
AP1	5′-GTCATACGACGGTACCTGCAGAATTCTC-3′	
RevKm2	5′-CATCACGACTGTGCTGGTCATTAAACG-3′	
2718REV2 (r)	5′-CGATGGACAACATTTGCATGG-3′	
2717REV2 (r)	5′-ATGGGCCGTAAAGCTTACC-3′	
2716REV2 (r)	5′-GCAACACTCTTAGAAATGTAACGC-3′	
2715REV2 (r)	5′-CACTTCGCAGCCAGCATGG-3′	
2714REV2 (r)	5′-GACTTGGTTGAACAGGTTGC-3′	
2717DIR1 (d)	5′-CGCGCAACGGGACTAACG-3′	
2716DIR1 (d)	5′-GGACTAGGAGCGACTGTGG-3′	
2715DIR1 (d)	5′-GCGTTTCAAGGCGATATATACC-3′	
2714DIR1 (d)	5′-AAGCTCGCCAGTGAAATTGG-3′	
2718REV1 (r)	5′-AGCTAGCGATCAGTAACACC-3′	
2717REV1 (r)	5′-TAAGGGTATGATTTATCGTCGC-3′	
2716REV1 (r)	5′-CGATACAGTATCAATTGCATCC-3′	
2715REV1 (r)	5′-TAAAAACTCTGAAATAGACGGCG-3′	
pEVS126Rev	5′-TCTCATCAACCGGAGCTCCCTCAC-3′	
pEVS126ForSacI	5′-TAACATCAGAGCTCTTGAGACACAACG-3′	
MM125R	5′-ATCAAGGAAAGGTACCAGATTAAGGGGTAG-3′	
MM126D	5′-ATGCTTAGGATCCAACTCTGAATTGTCCAC-3′	
MM127D	5′-GTCCAAACGGATCCAGCTTAATGAAAAT-3′	
MM146D	5′-GAGTATCTTGAGTACTGTTGCCTCAC-3′	
MM147R	5′-CGGTCTAGGTTAAAGTACTTCCTTCC-3′	

Mutant strains with deletion of CRISPR-Cas systems were constructed by allelic exchange mutagenesis using *sacB*/sucrose counter-selection as previously described^16,42^. The deletion in the genome was confirmed by PCR.

### Identification of the gene mutated in strain T102 by genome walking

Strain T102 was generated by transposon mutagenesis using plasmid pLOFKm that contains a mini-Tn*10* as previously described^30,43^. The genome walking technique was used to identify the unknown flanking genomic sequences adjacent to the site of transposon insertion^44^. Briefly, genomic DNA from T102, obtained using the CTAB method was digested with the blunt end restriction enzyme *Sca*I (Fermentas), which does not cut inside the transposon. Adaptors 1 and 2 and primer AP1, were designed based on the GenomeWalker Universal KIT (Clontech). In case we needed to clone the fragments after this protocol, Adaptor 1 primer was designed with the restriction sites *Eco*RI, *Xba*I, *Kpn*I and *Pst*I that do not cut inside the transposon, and *Hind*III and *Sma*I that cut in the mini-Tn*10* transposon. After the digestion of genomic DNA, Adaptor 2 primer was ligated to the 3′ ends of the fragments obtained, followed by ligation of Adaptor 1 primer to the free 5′ ends. After both ligations, double-strand DNA fragments have hanging 5′ ends. Next, a PCR was set with primers RevKm2, that binds to the insertion sequences at both ends of the transposon, and AP1, hybridizing the complementary sequence of the hanging fragment of Adaptor1. In the first PCR cycles, the polymerase only expands from RevKm2, as the 3′ ends of Adaptor2 do not reach the hanging fragment of Adaptor 1. Afterwards, the PCR proceeds from the generated sequences complementary to Adaptor1 using AP1 primer. In case that some PCR product with Adaptor 1 primer sequences at both extremes was generated, for example if a fail of the amination of Adaptor2 occurred, both ends of the fragment hybridize forming a “panhandle” structure that inhibits further amplification.

The application of this technique gave two bands for the genomic DNA digested with *Sca*I. One band would correspond to the sequence upstream from the transposon and the other band to the downstream sequence. Those bands were sequenced by the Área Científica y Técnica de Investigación (ACTI) of the University of Murcia, and blasted against the published genome sequence of *M. mediterranea*.

### Determination of the transcriptional unit including *ppoR*

In order to determine the genes in the transcriptional unit of the gene *ppoR*, interrupted by the transposon in strain T102, the RT-PCR technique was used. RNA was extracted from *M. mediterranea* MMB-1R using the RNeasy Midi Kit (Qiagen). Next, five different retrotranscriptions using SuperScript II RNase H reverse transcriptase (Invitrogen, CA) were set, using primers: 2718REV2 (hybridizes with MARME_RS14130 that encodes a diacylglycerol phosphate transferase, *pgsA*), 2717REV2 (hybridizes with MARME_RS14135, a gene similar to *uvrC*), 2716REV2 (hybridizes with MARME_RS14140, the gene mutated by the transposon in strain T102, named *ppoR*) and 2715REV2 and 2714REV2 that hybridize to the genes MARME_RS14145 and MARME_RS14150 coding for hypothetical proteins. With the cDNA fragments obtained, several PCR were set using primers 2717DIR1, 2716DIR1, 2715DIR1, 2714DIR1, 2718REV1, 2717REV1, 2716REV1 and 2715REV1.

### Enzymatic determinations

The enzymatic measurements of PPO and LOD activities were performed as previously described^23^.

### Complementation of the mutant strain PpoR^*−*^

The complementation of PpoR^−^ mutant strain was performed with the plasmid pEVS126SII, a derivative of pEVS126^45^ containing the gentamycin resistance gene of pBSL182^46^ instead of the native kanamycin selection. pEVS126SII was constructed by ligation of the *Sac*I digested Gm fragment from pBSL182 and the digested PCR product of pEVS126 with primers pEVS126ForSacI and pEVS126Rev. The MCS of pBSL182 was used to clone different versions of the *ppoR* operon. The plasmid containing the whole operon was constructed by PCR amplification from genomic DNA using primers MM125R and MM127D. The plasmid with only *ppoR* contains a *Kpn*I-*Bam*HI fragment of the PCR product MM125R-MM126D. The plasmid containing only the *uvrC* gene was PCR amplified from the plasmid containing the whole operon using primers MM146D and MM147R, which excise the *ppoR* gene leaving an intact native promoter region by *Sca*I digestion and religation. All the constructions were confirmed by PCR and their digestion pattern.

Next, all the plasmids were introduced into the PpoR^−^ mutant strain by conjugation with *E. coli* and selection in MMCRif50Km40Gm15 plates. The selected clones were checked by plasmid extraction and PCR.

### Phage quantification by the double layer agar technique

In order to quantify/test the sensitivity of different strains to phage CB5A, an assay using the double layer technique in MNG medium was performed as previously described^47^. The counts were referered to the counts on the wild type strain MMB-1R.

### Statistical analysis

The results of experiments with biological replicates are expressed as mean ± standard deviation. One-way ANOVA analysis of variance, followed by Tukey’s multiple comparison test, was used to assess the significance of differences between the samples analyzed.

### RNA isolation

RNA was isolated from different bacterial cultures using a single culture for each condition. On the one hand, strains MMB-1R and T103 at log phase in MNG medium at 25 °C and 130 rpm. On the other hand, strains MMB-1R, T102 and T013 incubated in MNGL medium in the same conditions. A sample of each culture was collected, and was inmediately mixed with double its volume of RNAprotect Bacteria Reagent (Qiagen) and incubated at room temperature for 5 min. After this incubation period, the samples were centrifuged at 5000×*g* for 10 min and the pellet was kept at − 20 °C until they were procesed. To extract the RNA from the samples, the RNeasy Midi Kit (Qiagen) was used with the optional on-column DNase digestion using the RNase-free DNase Set (Qiagen) according to the manufacturer’s specifications. All centrifugations of the columns were performed at 3850×*g* in a Hettich centrifuge with a swinging bucket rotor. The RNA was eventually eluted with RNase-free water. The RNA concentration and purity was analyzed in a 2100 Bioanalyzer (Agilent).

### RNASeq

#### Ribodepletion and cDNA library preparation

After total RNA sample QC, Illumina RiboZero kit was used to remove rRNAs. Subsequently a stranded cDNA library preparation from mRNA was performed using the Illumina TruSeq kit. The random hexamers were used and the chemically fragmented mRNA was reverse-transcribed to single stranded cDNAs following the kit protocols. The samples from cultures in MNGL medium were sequenced using single reads by BaseClear (https://www.baseclear.com/). Next, reads containing adapters and/or PhiX control signal were removed using an BaseClear in-house filtering protocol. The samples from cultures in MNG medium were sequenced using paired ends by NOVOGENE (https://en.novogene.com/). Raw reads were filtered as follows: (1) Reads with adaptor contamination were discarded. (2) The same for reads when uncertain nucleotides constituted more than 10% of either read (N > 10%). (3) When low quality nucleotides (base quality less than 20) constituted more than 50% of the read, it was also rejected. Only clean reads were used in the downstream analyses. All sequences have been deposited under accession number PRJNA676156.

#### RNASeq analysis

The MMB-1 reference genome was NC015276.1. The corresponding genome sequence (fasta) and annotation (gtf) files were downloaded from NCBI. The filtered reads were mapped to the reference genome using the Bowtie2 with the basic settings (-p 4 –fr ). The raw mapping file was sorted by samtools sort command to get the sorted file for the downstream analysis. HTSeq software was used to generate the raw counts table with the union mode. The CsrB and CsrC gene counts were manually added to the count table. CsrB gene count was acquired by samtools view -c on the sorted bam file with the positions 755,847–756,302. The CsrC gene count was acquired by samtools view -c on the sorted bam file with the positions 3,922,203–3,922,857. The Deseq2 normalized counts table were generated using Deseq2 package version v1.28.1 in R^48^. The normalized count table were used as the input for the downstream gene differential expression comparisons and data visualizations in this paper.

#### Coverage plot representation

To make the all bam files comparable to each other, we down-sampled each bam file to 1 million records by using samtools view -b -s seed [X] input.bam > output.bam, where the seed number used in our case is 333 and X is the size factors ( WT: 1e6/15,724,819 = 0.064; T103: 1e6/16,334,634 = 0.061), e.g. for WT, it is 333.064 as the format. The output down-sampled bam files were sorted and indexed by samtools for IGV visualization. For read depth calculation, we generated individual CsrB and CsrC bed files. Next, we used bedtools coverage -a CsrB/C.bed -b WT/T103.down-sampled.bam -d and redirected the command line outputs to a .txt file. The .txt files were then used as input and opened in Microsoft Excel for plotting the read depth at each nucleotide’s position.

## Supplementary Information

Supplementary Information.

## Data Availability

The RNAseq data generated and analyzed during the current study is available in the National Center for Biotechnology Information Sequence Read Archive (accession number PRJNA676156).
