# Supplementary material for: A histidine kinase and a response regulator provide phage resistance to Marinomonas mediterranea via CRISPR-Cas regulation
Source: Sci Rep. 2021 Oct 18;11:20564. doi: 10.1038/s41598-021-99740-9 (PMC8523701; doi:10.1038/s41598-021-99740-9)

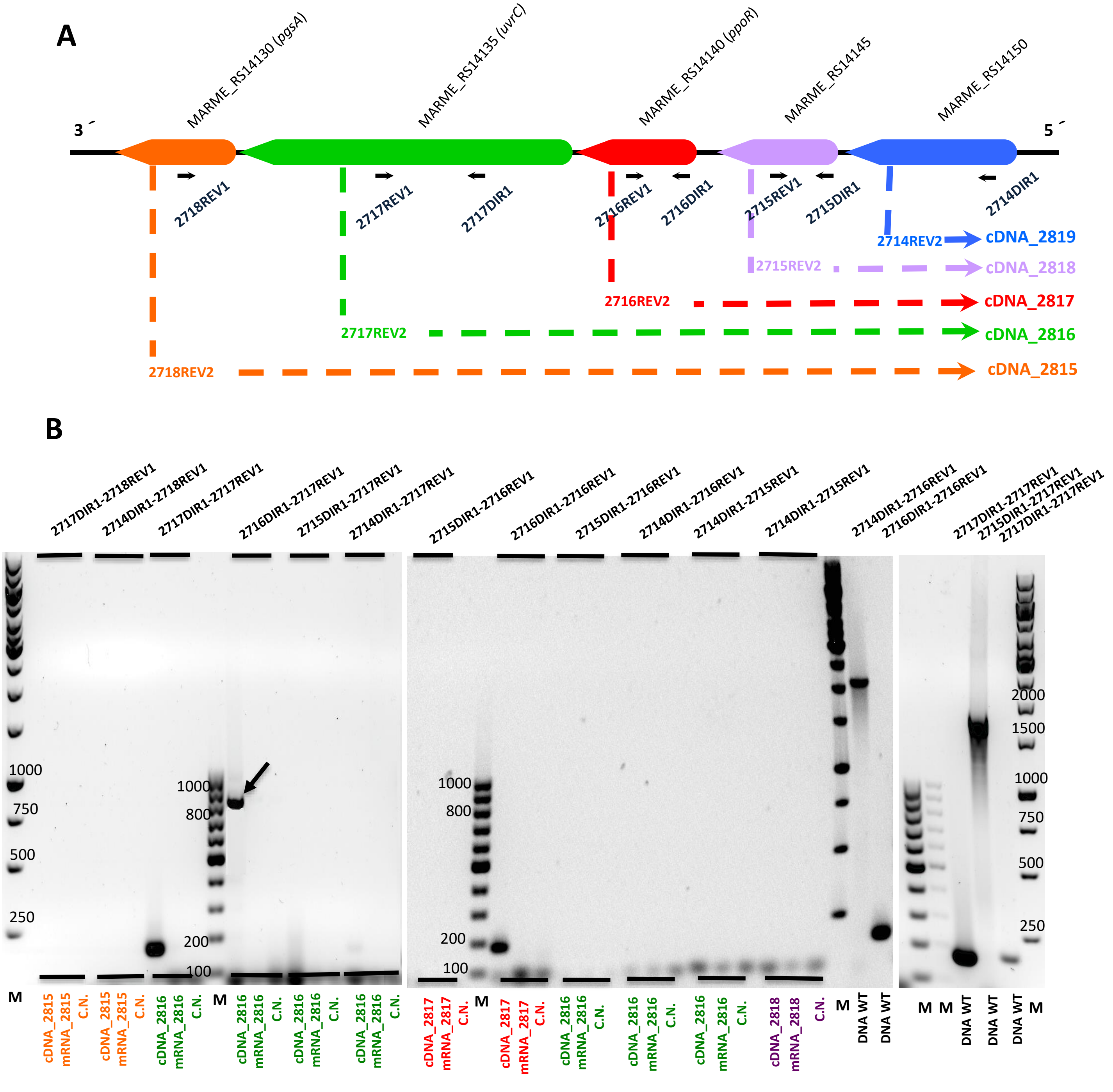

Supplementary figure S1. ***uvrC*** **and** ***ppoR*** **form part of the same transcriptional unit.** RT-PCR experiments were performed to identify genes in the same transcriptional unit as *ppoR*. **A**. Discontinuous lines represent the theoretical cDNAs of maximal length, in the case that all genes are cotranscribed, generated after different retrotranscriptions using primers (named as _REV2) in each gene. The location of the primers used for the PCRs aimed to detect in the cDNAs which genes form part of the same product of RT-PCR is also indicated. **B**. Agarose gels showing the PCR products obtained in the RT-PCR analysis. The pair of primers used are indicated on top of the gel. mRNA: control PCR using the mRNA to probe lack of DNA in the samples. C.N: PCR control for each primers pair without a template. DNA WT are controls for pair of primers using the genomic WT DNA, only some representative examples are shown. M denotes a lane with a ladder, including the band's size. The arrow indicates the amplified band that indicates that MARME_RS14140 and MARME_RS14135 belong to the same transcriptional unit. No evidences have been obtained of any other gene forming part of that unit, although additional experiments would be required to rule out completely that possibility.

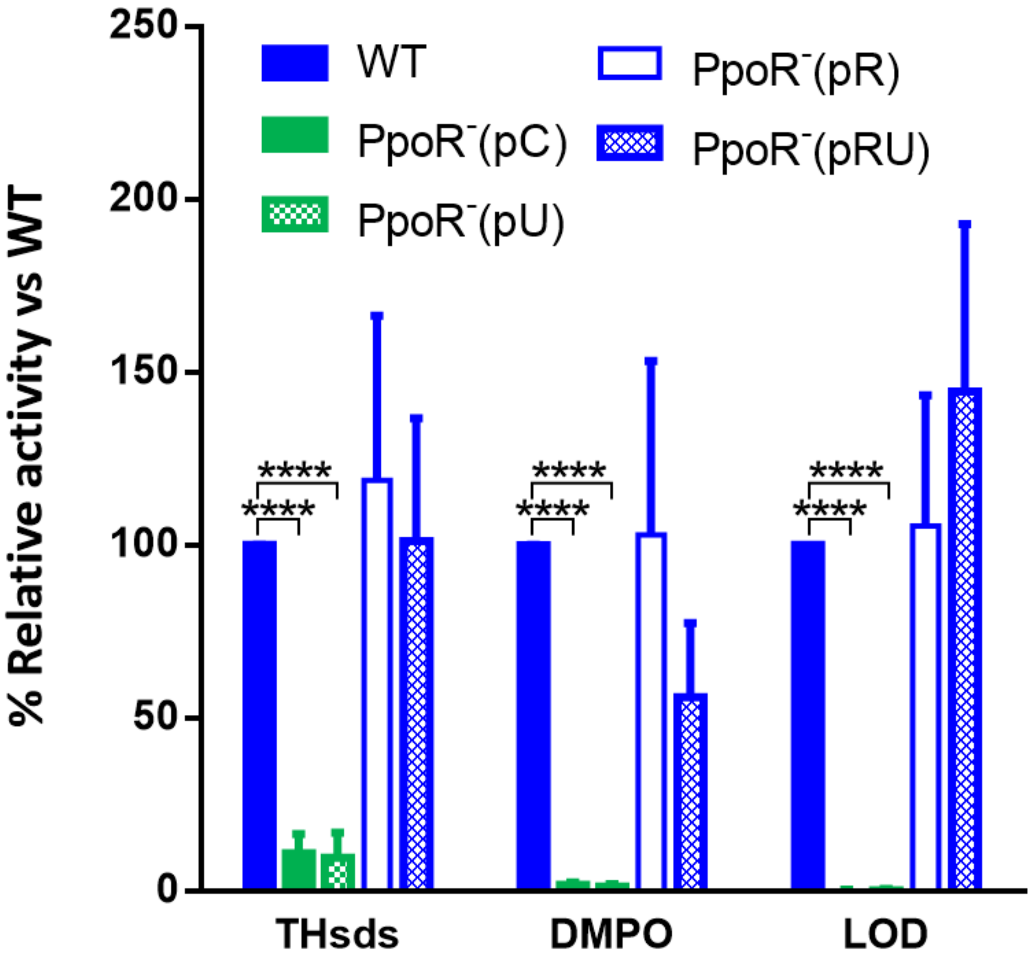

Supplementary figure S2. Complementation of the mutation in PpoR$^-$ strain by plasmids containing this gene. DMPO (Dimethoxyphenol oxidase activity, characteristic of laccase), THsds (Tyrosine hydroxylase activity in the presence of SDS, characteristic of the tyrosinase) and LOD (Lysine oxidase) were determined in strain WT (MMB-1R) and strain PpoR$^-$ (T102) transformed with different plasmids: pC, control plasmid pEVS126SII with no insert; pR, plasmid with p*poR;* pU plasmid with *uvrC*; pRU, plasmid with the operon *ppoR-uvrC*. Activities are expressed as average activities in the strains with the plasmids relative to the activities in the wild type strain plus standard deviation (n=3). ****P< 0.0001 according to the one way ANOVA analysis of variance, followed by Tukey's multiple comparison test used for statistical comparisons. No statistical differences were observed between the wild type strain and the strain PpoR$^-$ with plasmid pR or pRU.

Supplementary figure S3. Comparison of genes overexpressed (>two fold) (A) and repressed (< two folds) (B) in comparison with the wild type strain in strains PpoS$^-$ (magenta) and PpoR$^-$ (green) grown in MNGL medium.

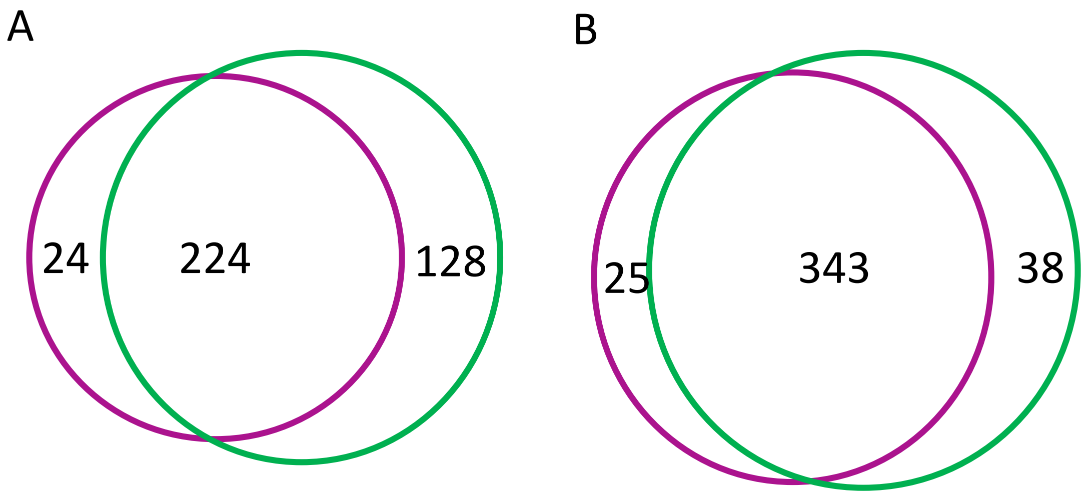

Supplementary figure S4. CsrB. A, Intergenic region between MARME_RS03610 and MARME_RS03615 (Range : 755858 to 756301). Between asterisk the predicted sRNA CsrB. In capital blue letters, the GacA box with the conserved residues underlined. Highlighted in yellow the palindromic sequences of the Rho transcriptional terminator. In red putative CsrA binding motifs, with those predicted in the RNA fold structure highlighted in blue. **B,** RNA fold structure determined using in the unafold web server the application "RNA Folding Form V2.3" (http://www.unafold.org/mfold/applications/rna-folding-form-v2.php/). The blue boxes show the predicted hairpin loops containing some of the CsrA binding domains.

A

ggctcagcgtgcttttgtccattattcatttttctCGTCAGAATATTCCTACAaag
tttgtcaggaaaatctctttttagcgtcgtctgttttcatctctttctgtttttta
ctttatccctttgtttataaggggattatctctttttttatgccttttccccttgtt
tgtttgtttttacaaaatcgcatatcttt*aattcatcgaaggatgaaacgggatc
tggcgataaggaattgcctgctagggaagcgctaattatttatggattcggctgga
agccaacagggagatggacgatacagtgcgatggattgtattgccacatggagtgg
gtaggactcgatagactatcgatactcaggaaagagccgttgtcaggatgacaaca
aaaacaagcgaccaaaaggtcgctttttttt*atgtcgaacgataatgcgctga

B

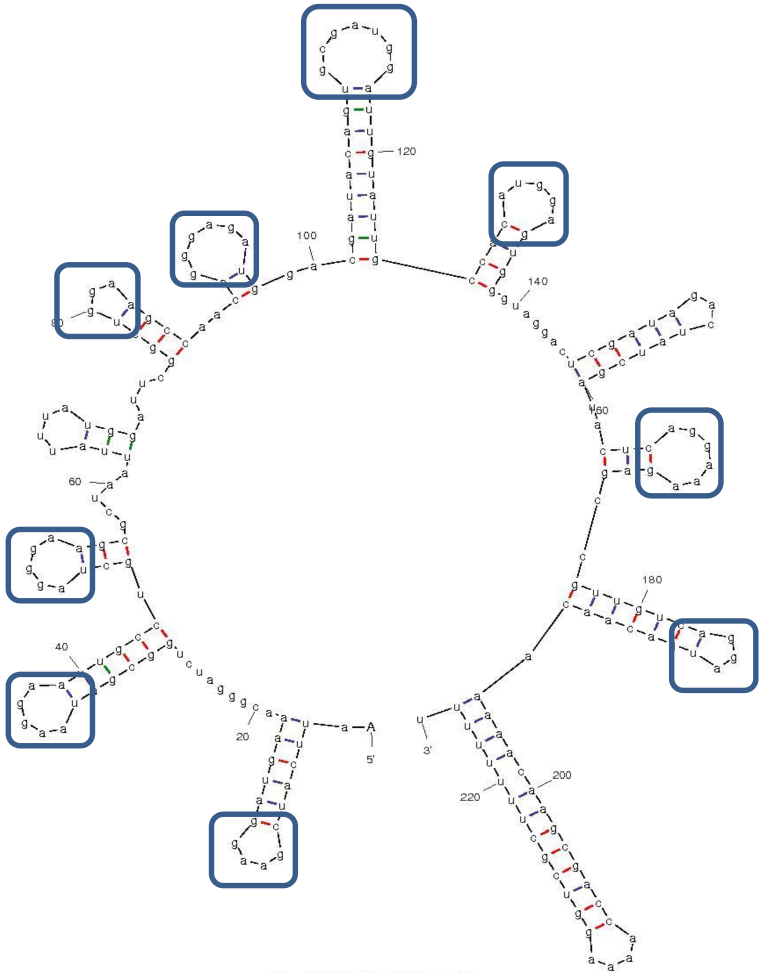

Supplementary figure S5. CsrC. A, Intergenic region upstream MARME_RS17890 (Range: 3922889 to 3922203). Between asterisk the predicted sRNA CsrC. In capital blue letters, the GacA box with the conserved residues underlined. Highlighted in yellow the palindromic sequences of the Rho transcriptional terminator. In red putative CsrA binding motifs, with those predicted in the RNA fold structure highlighted in blue. **B,** RNA fold structure determined using in the unafold web server the application “RNA Folding Form V2.3” (http://www.unafold.org/mfold/applications/rna-folding-form-v2.php/). The blue boxes show the predicted hairpin loops containing some of the CsrA binding domains.

A

ttttcgcgccgagccgcgattagttaaaagtcgTGTAGGGAAAATCTGACAgttcagtaagggat
atctgaatcacatgtagaaaactggaagtgtggtgcgtagttttattttaaattgttgatattta
aggaaaaaataaatattttctgtttttttatgaatttttacattcccaaataatagtgtacattt
*aacttatccaaacggacatcacggatgacggcacatgaagtgcagtatgacaggaaagtgatac
ggatacctaaggaatgagcaggatgctcaagctaaacggatttttctcacaggaagtggaatgac
gaaggaaagcgtcatgtgaccagggcggttggatctctaacggaagaaaggcaggaagctttact
catcggaagagtcgtttctaaggaaagaagcgagaaacggggtgacgtgagtcaccccttaatt
ctcagggaaaaataggatcttgggatataggctaacggaatgctaagagcatgaagctcgagaag
gatatcagcgtttgctggacaaggaatcaggaatgaagcgcttattggaatgagcgtcgctagga
tggcgagtaggattgaacactgttggatagagtgcacgtcaggaagacgagaaaataggcagcca
aatggctgctttttttt*gcgctaattttcgttcattat

B

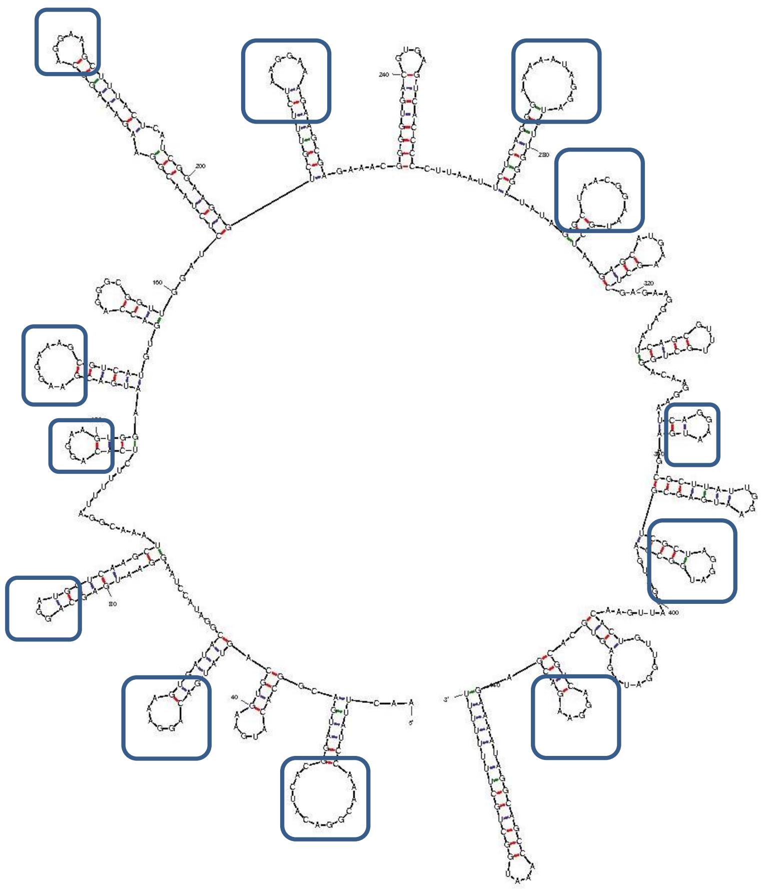

Supplementary figure S6. Evidences of the transcriptional expression of *csrC* (top) and *crsC* (bottom). Taking into consideration the predicted annotation and gene coverage from RNA sequencing results, the starting and ending positions were refined to be 756058-756260 (+) for *csrB* and 3922251-3922704 (-) for *csrC* genes and used in the coverage plot and IGV plot.

A. **Coverage plot showing the transcriptional activity of the sRNAs detected in this study.** The coverage for the wild type strain (blue) and the PpoS⁻ mutant (orange) are shown.
B. **IGV plot of the expression of the sRNAs and surrounding genes.** The *csrB* and *csrC* annotations were manually added in the NC_015276 genome based on the bioinformatic predictions.

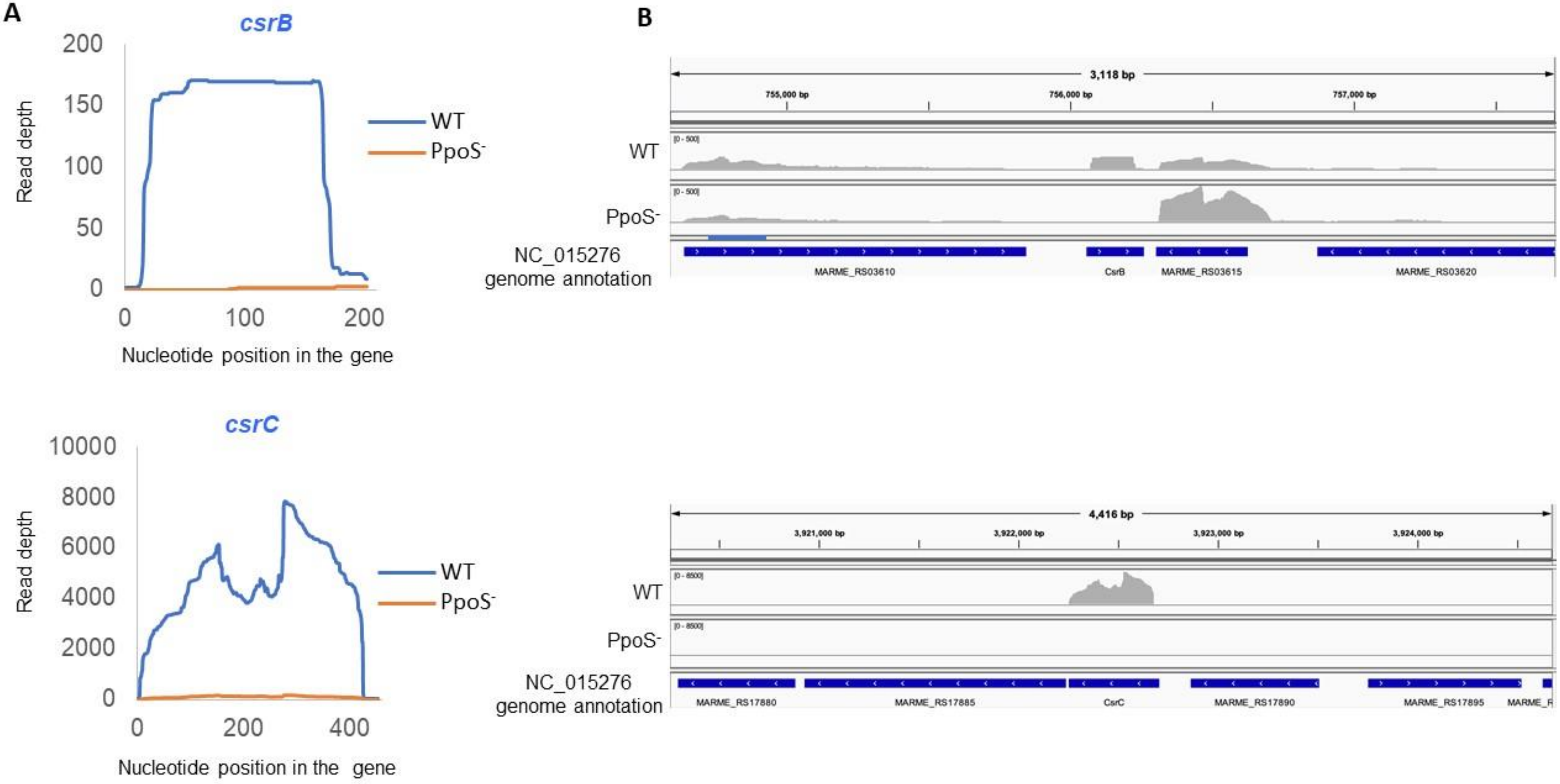

Supplementary figure S7. Putative CsrA binding sites close to CRISPR-Cas systems in *Marinomonas mediterranea*. **A**, I-F system with the CsrA binding site marked by a red star. The two genes upstream the I-F system where the target is located code for a methyl transferase domain containing protein and a WYL domain containing protein, respectively. **B**, Sequence of the CsrA binding site in the I-F system with the conserved motifs marked in blue. **C**, III-B system with the CsrA binding site marked by a red star. **D**, Sequence of the CsrA binding site in the III-B system with the conserved motif marked in blue. Arrows indicate the leader sequence of the CRISPR arrays. The numbers on top of the diagrams in the CRISPR systems are the MARME_RS locus tag of each gene.

A

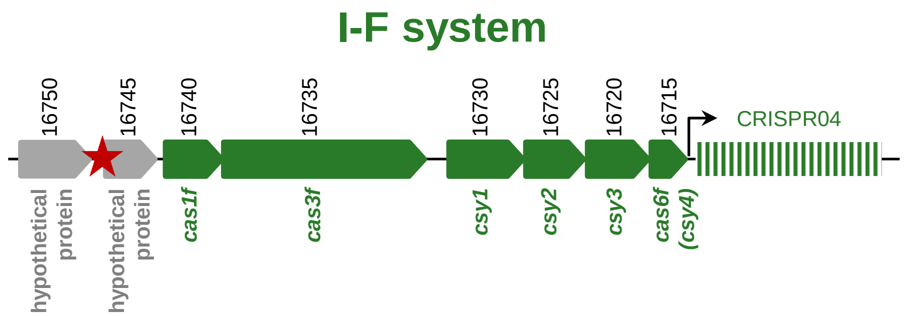

B

>MARME RS16745, upstream from -200 to +30
CGACCTCTTTTCTCTTCCATTTTACAAATCCGAACTTGAGTTCCGTATAACGC
ATCCTTTACAGTGTTGTCTCCTTAAAGAAGGTACTTTAAAGAAGACACTTTAA
AGAAAACACCTAAAAAGCGTTAAAGGGCAGCCTAAATACAGGAAGCGTTTACA
GAAGTCGTCAAATAAGAGGCGGCGACTAAAAAGGAGCGTAAATGGAAGACAAA
GCGGCAGGACATTTAAAAC

C

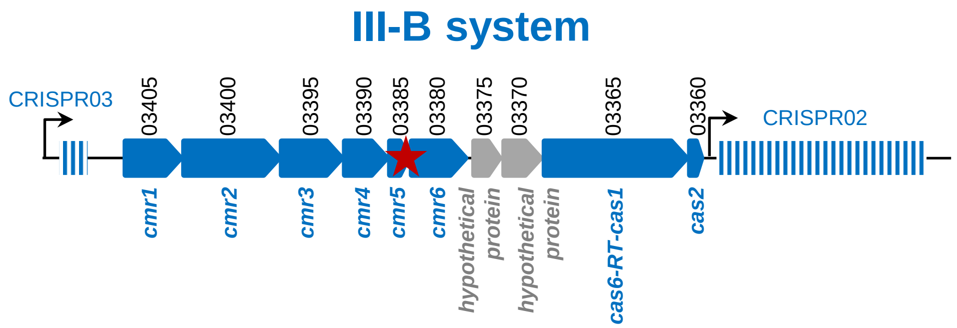

D

>MARME_RS03380, upstream from -200 to +30
AATAGCGGCCACCTAGAAAACCAGAATACGCCAAACTCAGACAACGGCAAAG
AGCTGCACCAGCAAATCATTCAGTCTGATATGACAAAATTCATGCAGTTAAC
TCAACAATCCCTCGATGCCAGTGGAGCACTTCGTCGCTATGTTCAAGGTTTA
ATGGACACAAACAAAAAGGATGATGATTCGCAGGAGGAAACCTTATGAGCTC
TGTTCAATTAGTTCCCCAGAGAT

Supplementary figure S8. Regulation of CRISPR-Cas expression in *M. mediterranea* by the histidine kinase PpoS and the response regulator PpoR. Transcriptomic levels, expressed as normalized counts, of the I-F (**A**) and III-B (**B**) CRISPR-Cas systems in different genetic backgrounds and MNGL or MNG media. The t-Student test was applied to compare the levels in WT strains (n=2) with the levels in the mutant strains. (n=3). *P< 0.05; **P< 0.01. ***P< 0.001, ****P< 0.0001, "ns" stands for not significant.

A

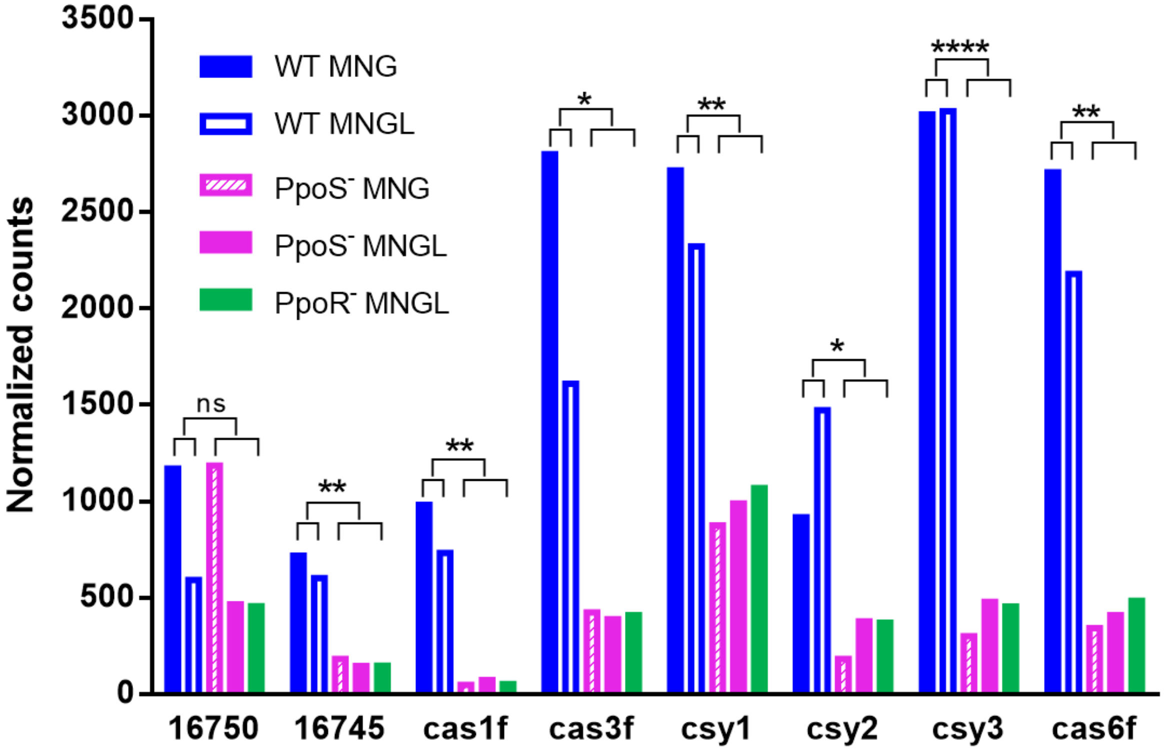

B

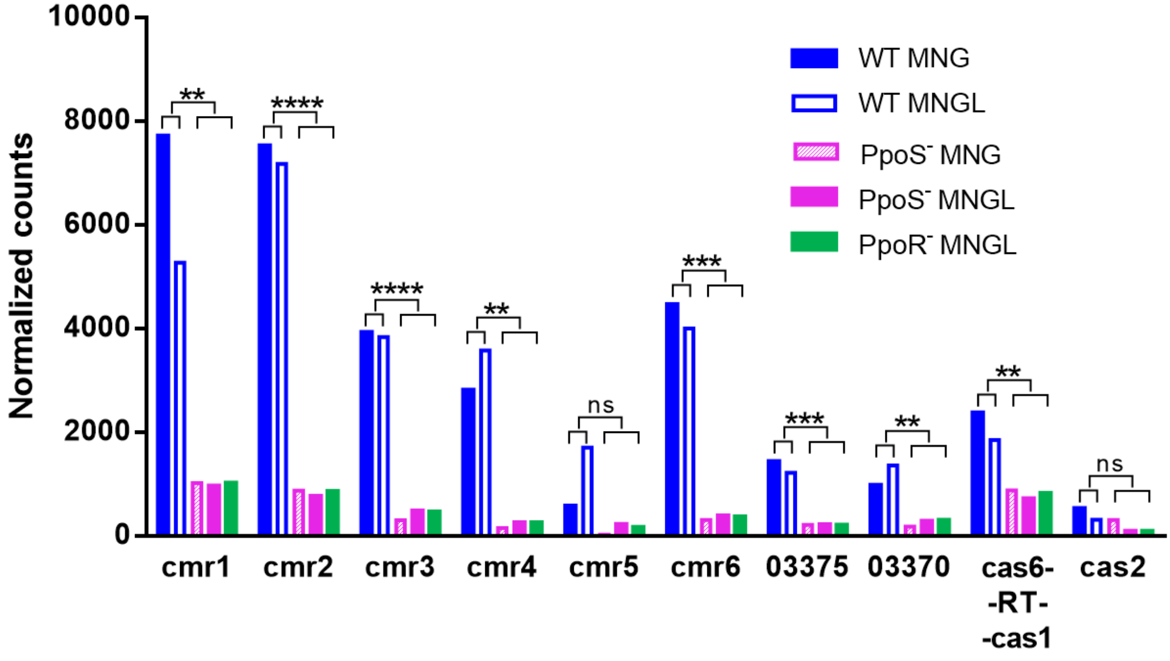

Supplement: Supplementary file 1 — Supplementary Information. [file 41598_2021_99740_MOESM1_ESM.pdf]
